# Latest Insights on Novel Deep Eutectic Solvents (DES) for Sustainable Extraction of Phenolic Compounds from Natural Sources

**DOI:** 10.3390/molecules26165037

**Published:** 2021-08-19

**Authors:** Julio Serna-Vázquez, Mohd Zamidi Ahmad, Grzegorz Boczkaj, Roberto Castro-Muñoz

**Affiliations:** 1 Tecnologico de Monterrey, Campus Ciudad de México, Calle del Puente 222, Ejidos de Huipulco, Ciudad de México 14380, Mexico; juliosernav@outlook.es; 2 Organic Materials Innovation Center (OMIC), Department of Chemistry, University of Manchester, Oxford Road, Manchester M13 9PL, UK; mohdzamidi.ahmad@manchester.ac.uk; 3 Department of Process Engineering and Chemical Technology, Faculty of Chemistry, Gdansk University of Technology, 11/12 Narutowicza St., 80-233 Gdansk, Poland; grzegorz.boczkaj@pg.edu.pl; 4 Tecnologico de Monterrey, Campus Toluca, Av. Eduardo Monroy Cárdenas 2000 San Antonio Buenavista, Toluca de Lerdo 50110, Mexico

**Keywords:** green solvents, biologically active compounds, selective separation, medicinal plants, ultrasonic-assisted extraction, microwave-assisted extraction

## Abstract

Phenolic compounds have long been of great importance in the pharmaceutical, food, and cosmetic industries. Unfortunately, conventional extraction procedures have a high cost and are time consuming, and the solvents used can represent a safety risk for operators, consumers, and the environment. Deep eutectic solvents (DESs) are green alternatives for extraction processes, given their low or non-toxicity, biodegradability, and reusability. This review discusses the latest research (in the last two years) employing DESs for phenolic extraction, solvent components, extraction yields, extraction method characteristics, and reviewing the phenolic sources (natural products, by-products, wastes, etc.). This work also analyzes and discusses the most relevant DES-based studies for phenolic extraction from natural sources, their extraction strategies using DESs, their molecular mechanisms, and potential applications.

## 1. Introduction

Since the beginnings of humankind, medicines have been a crucial part of our progress as a species. The search for remedies against diseases probably started with plants, as evidenced by archaeological artifacts that demonstrated the use of medicinal plants during the Paleolithic. Furthermore, the oldest written evidence was found on a Sumerian clay slab, dating back to approximately 3000 B.C. Many medicinal plant benefits are attributed to phenolic compounds, given their antioxidant, anticancer, antibiotic, antifungal, and anti-inflammatory activities [1,2]. Phenolic compounds contain at least one aromatic ring with one or more hydroxyl groups. They play an essential role in plant growth, reproduction, and protection against parasitoids, pathogens, and predators [3,4,5]. There are more than 8000 plant phenolic compounds, and their extraction from natural sources is of high interest in the industry due to their use in pharmaceuticals, beverages, food, and cosmetics [6,7].

Typically, phenolic compounds are extracted using organic solvents (e.g., methanol, ethanol, acetone, ethyl acetate, hexane, benzene, etc.) or petroleum-based solvents [8,9]. Nevertheless, due to environmental, health, and safety concerns, a large amount of research has been done in order to synthesize safer extraction solvents from renewable sources [10]. Ionic liquids (ILs) are presented as an alternative to the solvents described above, given their notable attributes, such as high thermal and chemical stability, non-flammability, and low vapor pressure [11]. However, their high cost, toxicity, dangerous decomposition by-products, and poor biodegradation levels are matters of concern, and given that some of the most used ILs constituents (i.e., imidazolium, pyridinium) are petroleum-derived, further research on alternative solvents has been carried out, leading to the creation of deep eutectic solvents (DES) [12].

The term DES was first introduced in 2003 by Andrew P. Abbott and his group when creating eutectic mixtures of urea and quaternary ammonium salts that could be used as solvents at room temperature as a result of hydrogen bonding between the species that make up these solvents [13]. Throughout the years, DESs have been catalogued as great substitutes for conventional solvents considering their similar physical properties to ILs, presenting advantageous characteristics such as low cost, biodegradability, non-toxicity, low volatility, easy preparation, and even biocompatibility [14,15]. Additionally, phenolic stability in DESs has been reported [16,17]; hydrogen bonding between DESs and phenolic compounds prevents degradation by reducing the phenolic molecules’ movement, thus decreasing their contact time with air and simultaneously avoiding oxidative degradation [18]. Furthermore, DESs can be designed for highly efficient extraction of specific compounds improving their bioactivity and stability compared to traditional solvents [19].

Given the high value of several phenolic compounds for pharmaceutical and food industries, particular interest is shown in DES-based extraction methods since various DES-constituting compounds are approved for human consumption as food additives or supplements. This is the case of choline chloride (ChCl), the most widely studied hydrogen bond acceptor (HBA) in DESs [20,21], as well as other components including 1,2-propanediol [22], organic acids, such as acetic, citric, lactic, malic, and tartaric acids [23]; sugars like fructose, glucose, sucrose, ribose, and mannose [24,25,26]; and amino acids like l-proline and l-alanine [27,28,29].

**Figure 1:**
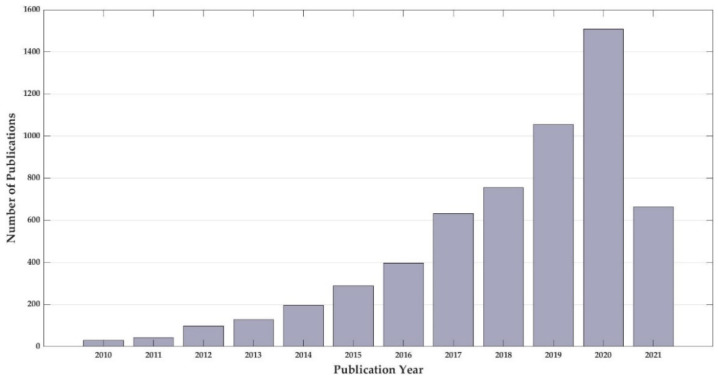
Number of publications that include the term “deep eutectic solvent” in their title since 2010. Source: Web of Science, 12 June 2021 [40].

DES importance also relies on their wide application range, including analytical chemistry, biomolecule extraction, biocatalysis, biomass processing, biodiesel and gas separation, metallurgy, nanomaterials synthesis, therapeutics, food and water sample analysis [10,15,30,31,32,33,34,35,36]. Their high potential results from almost unlimited possible chemical combinations for their synthesis. This way, a fit-to-purpose DES with unique selectivity can be synthesized [37,38,39]. DES research has had substantial growth throughout the years (illustrated in Figure 1), owing to its remarkable properties and its large application field, including phenolic compounds extraction from natural sources. 

This review summarizes the most relevant studies in the last year involving phenolic compounds extraction from natural sources through different DES-based extraction methods, where molecular mechanisms involved in each extraction procedure are discussed. This work also aims to exhibit the current DES research and its potential use in various industries.

## 2. Principles of DES

DESs are mixtures of two or more compounds that present a significantly lower eutectic point than that of the ideal liquid mixture, as well as being liquid at ambient temperatures [41]. These solvents consist of a compound acting as a hydrogen bond donor (HBD) and a compound that acts as HBA [30,42]. DESs, similar to other eutectic mixture, possess a lower melting point than their components in their pure form [43,44]. The two most studied DES subgroups are natural deep eutectic solvents (NADES), which are based on sugars, alcohols, organic acids, amino acids, or amines [45], and therapeutic deep eutectic solvents (THEDES), where one or more of their components is an active pharmaceutical ingredient [46]. NADESs have been indicated as possible solvents present in living cells, thus explaining the presence of compounds at much higher concentrations than what is soluble in aqueous solutions [47]. Furthermore, NADESs reduce physicochemical constraints of metabolites transport and cellular processes through the formation of liquid microenvironments [14].

DES composition can be described with the general formula, as follows:

Cat^+^X^−^zY, (1)

Cat^+^ is an ammonium, phosphonium, or sulfonium cation; X^−^ is a Lewis base, commonly a halide anion; Y is a Lewis or Brønsted acid, and z represents the number of Y molecules interacting with X^−^, the anion [48,49].

As reported in Table 1, Abbot’s group have classified DES into four different types [48,50].

Type III DESs are the most studied and are commonly based on ChCl, an extensively used HBA for its low cost, non-toxicity, and biodegradability [49]. Type III HBDs are generally alcohols, amides, amino acids, carboxylic acids, and sugars [49,51].

The two most used methods in DES preparation are (1) grinding and (2) mixing and heating, preferred due to their simplicity. Grinding involves the use of a mortar and pestle where the DES components are triturated at room temperature until a homogeneous solution is formed. Mixing and heating is carried out in a container under constant stirring at a set temperature (usually between 50 and 100 °C). The DES precursors are mixed until the eutectic solution is formed [49]. Other preparation methods are freeze-drying and evaporation [11,52]. Several DES-based extraction methods have been reported; in fact, many of them are well-established methods that have been operated with conventional solvents, now adapted for DES uses. Several of the most reported methods include ultrasound-assisted extraction (UAE) and microwave-assisted extraction (MAE); however, traditional heating and stirring continues to be employed because of its simplicity and low cost. 

DES viscosity is an important property that affects extraction efficiency as lower viscosities are correlated with a higher mass transfer and extraction yield [53]. Essentially, the two options to reduce viscosity are water addition or changing one or more of the solvent components [20]. Through the hole theory, Abbot et al. [54] concluded that high viscosity is attributed to solvent species having a larger molecular radius compared to the average void radius. This hindrance can be overcome by using smaller cations or fluorinated HBDs [54]. Viscosity reduction through co-solvents addition (e.g., water) is commonly used for mass transfer enhancement. Water increases solvent polarity, which might be beneficial for certain compound extraction, depending on their polarities. However, water addition can result in loss of the DES molecular structure caused by the weakening of intramolecular interactions between the DES components [43].

**Figure 2:**
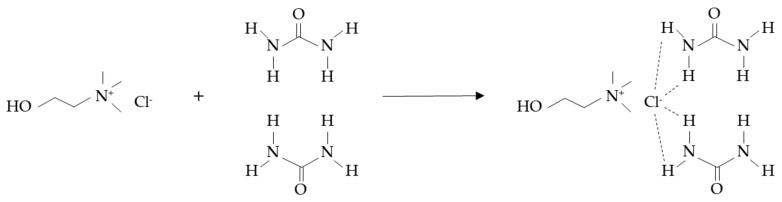
Hydrogen bonding in a ChCl:urea DES.

Hydrogen bond strength between the DES components and cation symmetry are key parameters in DES synthesis. The most significant freezing point depressions are observed in those mixtures where the DES components have a higher ability to form hydrogen bonds and a lower cation symmetry [55]. Other factors affecting DESs physicochemical characteristics are the charge delocalization process from hydrogen bonding formation, the electron density in hydrogen bond networks, and the presence or absence of functional groups, leading to different supramolecular structures with different melting points [56]. A schematic diagram of hydrogen bonding in a ChCl:urea DES is presented in Figure 2.

## 3. Phenolic Compound Extraction from Natural Sources

The section presents the studies reported on DES-based phenolic extraction from natural sources, emphasizing their molecular mechanisms, characteristics and advantages of each extraction process. Table 2 summarizes the sources, DES components and their molar ratio, co-solvent addition, extraction method, and extraction yield of each study.

Phenolic compound extraction from natural sources is necessary to obtain high-added value compounds that are crucial feedstock in the manufacture of several pharmaceutical, cosmetic, food, and nutraceutical products [81]. Manuka, the most widespread native shrub in New Zealand, possesses great importance in the country since it has long been used as a traditional medicine with antibacterial and antioxidant properties associated with its high phenolic content, and nowadays, it is a notable source of honey and essential oils. Alsaud et al. [57] studied phenolic extraction from manuka (*Leptospermum scoparium*) leaves using eight different DESs, constituted by either ChCl or tetrabutylammonium chloride (TBAC) as the HBA, combined with either ethylene glycol, 1,3-propanediol, acetic acid, or lactic acid as the HBD. The DESs were synthesized by stirring at 80 °C. Extraction was performed in a stirred vessel at room temperature for 1 h, with 5% biomass. The obtained extraction yields with different DESs were compared with the one achieved with ethanol as the extraction solvent; TPC (Total phenolic content) of 45.04 mg GAE g^−1^ (GAE = Gallic Acid Equivalents). Three of the DESs, ChCl:ethylene glycol (molar ratio of 1:2), ChCl:lactic acid (1:2), and ChCl:1,3-propanediol (1:3), showed higher extraction efficiencies than ethanol by presenting a final yield of 56.87, 52.51, and 50.67 mg GAE g^−1^, respectively. The high efficiency of ChCl:ethylene glycol and ChCl:1,3-propanediol DESs was attributed to their lower viscosity. However, the superior outcome of ethylene glycol-based DES was ascribed to its ability to form multiple hydrogen bonds with phenolic compounds and be only a two-carbon chain-length, which is believed to interact more easily than other polyalcohols [82]. Regarding the carboxylic acid-based DESs, the more viscous lactic acid DES presented a higher extraction efficiency than using acetic acid solvent, accredited to its higher number of OH groups. Using the ChCl:lactic acid DES, the authors reported the optimal extraction conditions at 50 °C, extraction duration of 1.07 h using 5.07% biomass, where they successfully extracted 59.82 mg GAE g^−1^. Additionally, the extracted phenolic compounds presented high stability in DES, even after eight days in storage.

Resveratrol is a phenolic compound widely used in the pharmaceutical and food industry due to its anti-inflammatory, anti-oxidative and anti-viral properties [83]. A high level of resveratrol is found in *Polygonum cuspidatum*, particularly in the root. However, it is majorly present as polydatin, its glycoside form [84], consequently, more efficacious methods of extraction and conversion of polydatin to resveratrol have been searched to upgrade or replace traditional methods, which are either costly, time-consuming, non-efficient, or harmful to the environment. Recently, Sun et al. [58] developed a one-pot polydatin-to-resveratrol extraction and conversion method from *Polygonum cuspidatum* root using eleven DESs. The method consists of stirring (at 500 rpm) the plant-DES mixture tube (with 4.5% HCl) in an oil bath at 80 °C for 80 min. Five out of the eleven DESs (tetraethylammonium chloride(TEA):ethylene glycol (1:2), TBAC:ethylene glycol (1:3), TEA:glycerin (1:2), TEA:1,4-butanediol (1:2), and triethyl benzyl ammonium chloride (TEBAC):1,4-butanediol (1:2)) presented higher resveratrol extraction yields than methanol and ethanol, tested at similar test conditions. The highest extraction efficiency was obtained with TBAC:ethylene glycol DES (9.00 ± 0.28 mg/g), attributed to its low viscosity, low pH, and a stronger hydrophobic interaction between the solvent and resveratrol. The optimized extraction parameters, determined using the TBAC:ethylene glycol solvent, are: stirring at 80 °C for 80 min in a 40% (*v/v*) water in DES, 4.5% (*m/v*) HCl, 40:1 liquid–solid ratio. The suggested method was compared against UAE and produced a higher resveratrol yield even though the presence of cavitation facilitates interaction between the solvent and the plant, thus proving the effectiveness of the stirring step. Contrarily, UAE could not penetrate the plant powder deposited at the bottom of the tube, leading to unextracted or unconverted compounds. With these optimum conditions, the extraction–conversion process reached a resveratrol yield of 12.26 ± 0.14 mg g^−1^.

Torres-Vega et al. [59] reported polyphenols extraction from *Buddleja globosa* Hope leaves through heating and mixing with DESs. *Buddleja globosa* is a medicinal plant traditionally used by the Mapuche for the treatment of wounds and dermatological, gastrointestinal, and hepatic disorders [85,86]. Water was added to all the prepared DESs, producing 20% water aqueous solvents, and the extractions were carried out at 60 °C and 340 rpm stirred for 50 min with 0.1 g plant in 10 mL solvent. Eight DESs were used; however, only five were suitable for individual phenolic quantification given the poor separation of forsythoside B and verbascoside in the other three. As for total phenolic extraction, only the ChCl:1,2-propanediol (1:3) solvent presented a significantly lower yield than 80% compared with methanol under the same extraction conditions. Whereas, the lactic acid:glycerol:water (3:1:3) and ChCl:glycerol (1:2) solvents did not show significant differences with the methanol extraction yield. Nevertheless, the other two DESs, ChCl:lactic acid (1:1) and l-proline:citric acid (1:1), displayed a significantly better total phenolic recovery than methanol. All five solvents showed significantly higher luteolin 7-*O*-glucoside extraction yields than methanol extraction, similarly for verbascoside yields except for the ChCl:1,2-propanediol solvent. This solvent exhibited the highest luteolin 7-*O*-glucoside extraction yield but the lowest for verbacoside and total phenolic yields. The trend is due to ChCl:1,2-propanediol’s higher extraction affinity towards flavonoids, similarly reported in other studies [87]. The poor extraction of the other phenolic compounds is probably due to its polarity. Using this new procedure, it was concluded that the ChCl:lactic acid and l-proline:citric acid DES solvents presented the highest phenolic extraction capacities from *Buddleja globosa* and are promising alternatives to traditional solvents.

Wojeicchowski et al. [60] screened ten DESs to extract phenolic compounds from rosemary (*Rosmarinus officinalis* L.) leaves. The extraction was done by heating and stirring at 30 °C and 600 rpm for 15 min with a liquid-solid ratio of 20:1. All DESs were prepared with ChCl as the HBA with various HBDs including acetic acid (1:2), lactic acid (1:2), oxalic acid (1:1), 1,2-propanediol (1:2), ethylene glycol (1:2), glycerol (1:2), xylitol (2:1), sorbitol (2:1), xylose (2:1), and zinc chloride (1:2). The extraction performances of pure DESs and aqueous DESs (30% water) were compared to pure ethanol and 70% ethanol. From the ten pure DESs, phenolic content was not observed in six of them, associated with their high viscosities. Meanwhile, the ten aqueous DESs showed higher extraction yields than 70% ethanol and significantly lower extraction yields than pure ethanol. The extraction optimization was conducted using the best DES (ChCl:1,2-propanediol) and was determined to be at 65 °C, with a 40:1 liquid-solid ratio with a 50% aqueous DES, resulting in the highest total phenolic yield of 78 mg GAE g^−1^. 

Đorđević et al. [61] reported total phenolic and total flavonoid extractions from ground black mustard (*Brassica nigra* L.) seed using triethanolamine (TEOA):glycerol, TEOA:propylene glycol, and ChCl:urea DESs. The DESs were prepared with a molar ratio of 1:2, along with DES mixtures with 25% water or ethanol. Extraction was performed through heating at 65 °C for 3 h and stirring at 1000 rpm, and a liquid–solid ratio of 10:1 (v/w). Out of the three pure DESs, the highest total phenolic extraction was obtained with the TEOA:propylene glycol solvent, providing 23.5 ± 0.7 mg of GAE g^−1^. Nevertheless, the highest yield was obtained with a mixture of TEOA:glycerol with 25% ethanol, producing 32.2 ± 0.2 mg of GAE g^−1^. Overall, it was observed that the addition of ethanol or water to each DES increased the extracted phenolic content, owing to the reduced solvent viscosity that simultaneously enhanced the phenolic transfer between the seeds and the DES [12,88]. However, the TEOA:propylene glycol-water mixture behaved differently. It displayed a reduced extraction yield, which is attributed to DES nanostructure debilitation or disintegration due to water-DES interaction, resulting in a decline in solvent extraction capability [3,43,89]. Regarding the total flavonoids content (TFC), the TEOA:propylene glycol with 25% ethanol showed the highest yield at 7.4 ± 0.3 mg of QE (quercetin equivalents) g^−1^. The higher phenolic and flavonoid recoveries using ethanol-DES mixtures were attributed to an increase in selectivity due to their polarity.

**Figure 3:**
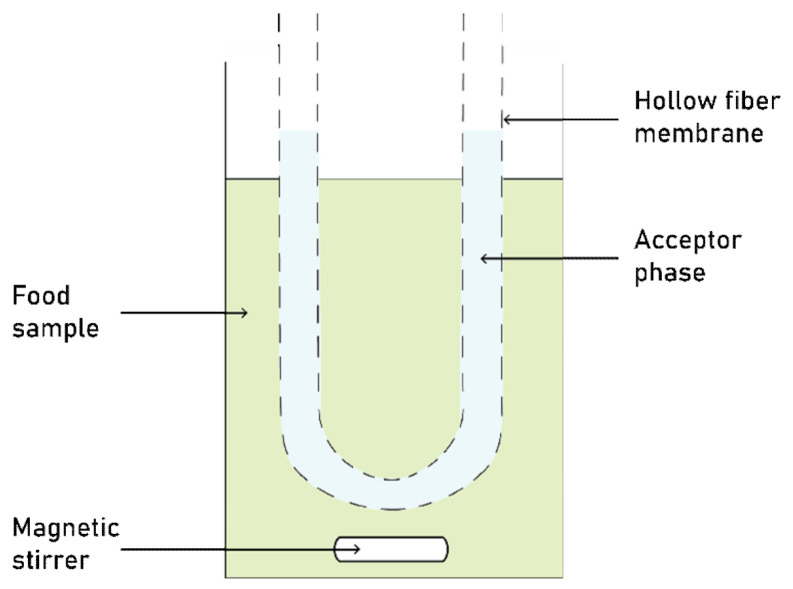
Two-phase hollow fiber-liquid microextraction setup. Adapted from Esrafili et al. [93].

Nia and Hadjmohammadi [62] performed caffeic acid extraction from coffee, green tea, and tomato through a two-phase hollow fiber-liquid microextraction (HF-LPME) using a hydrophobic DES as an acceptor phase. The two-phase HF-LPME consisted of a porous polypropylene hollow fiber filled with an acceptor solution, immersed in an immiscible solution of an aqueous sample and donor phase (see Figure 3). The target analytes in the aqueous sample were extracted into the liquid-supported membrane. The solvent selection for acceptor solution is crucial for an adequate extraction performance, as the solvent must be immiscible with the donor phase and have a high affinity with both the hollow fiber and the target compound. Conventional organic solvents are typically used for extraction; nevertheless, they present significant drawbacks such as instability, toxicity and volatility. More advanced solvents such as ionic liquids have also been used as the acceptor phase; however, their anions can hydrolyze into toxic species, in addition to their readily high cost and time-consuming synthesis. Hence, DESs are the obvious solvents of choice and have been proposed for HF-LPME [90,91,92]. For this study, three DESs were prepared: l-leucine:lactic acid, l-arginine:lactic acid, and l-serine:lactic acid, each at 1:3, 1:4, and 1:5 molar ratios. Based on the physicochemical properties of the produced DESs, the 1:4 molar ratio l-serine:lactic acid solvent was selected due to its high capacity to form π-type hydrogen bonds between the caffeic acid’s conjugated aromatic rings and the solvent’s polar groups. Moreover, to further reduce the DES viscosity, 30% methanol was added as a co-solvent. In the experiment, the hollow fiber was first immersed into the DES solvent for 30 s, followed by a 50 µL DES addition into the hollow fiber lumen side. Subsequently, the DES-supported hollow fiber was placed into the sample solution and stirred. The caffeic acid extraction yields from tomato, green tea, and coffee sample obtained were 0.032, 0.022, and 0.012 µg g^−1^, respectively. 

Cui et al. [63] reported a phenolic extraction from green tea by heating and stirring with five different DESs: ChCl-based DES with ethylene glycol, glycerol, glucose oxalic acid, and citric acid as the HBDs. The ChCl:ethylene glycol (1:2) was the best performing DES, attributed to its strongest affinity to phenolic compared to the other DESs, due to the good combination of a strong HBA and a relatively weaker HBD. The extraction produced a maximum yield of 20.12%, conducted with a liquid-solid ratio of 44:1 at 84 °C for 39 min. 

Huang et al. [64] extracted anthraquinones from Radix et Rhizoma Rhei, a Chinese medicinal plant from the *Polygonaceae* family, using novel hydrophobic DESs. The plant was used because hydrophobic anthraquinones (e.g., aloe-emodin, emodin, chrysophanol, physcion, and rehin) are one of its main chemical components [94]. Different alkanols (n-octanol, n-decanol, 1-dodecanol, and 1-tetradecanol) and alkyl carboxylic acids (n-octanoic acid, decanoic acid, and 10-undecenoic acid) were used for the synthesis of nine green DES solvents. In general, these DESs presented low viscosities ranging from 4.03 to 12.26 mPa·s, suitable for a high mass transfer between the plant and the solvent due to the absence of coulombic forces [51]. Additionally, these DESs were mixed with water at different volumetric ratios, confirming their immiscibility and thus their hydrophobicity. Based on the physiochemical analysis and protocol optimization, the 1-tetradecanol:10-undecenoic acid (1:4) presented the best dissolution performances and was selected for the extraction process. Before the extraction, a pectinase solution was used for plant cell walls impairment and then deactivated by heat. Later, 385 μL of concentrated HCl were added to obtain a 10% *w/v* HCl solution. For the extraction, DES and plant material were mixed in the aqueous solution at a 12:1 volume-to-mass ratio (mL g^−1^) at 67 °C for 20 min under 500 rpm stirring. After that, the solution was centrifuged to collect the anthraquinones in the DES phase and quantified by HPLC with diode array detection. Finally, the yields for four different samples from different Chinese provinces were obtained, and the sample from Sichuan presented the highest total anthraquinones extraction yield (ca. 21.52 mg g^−1^). The yields were comparable to values given for anthraquinones extraction from Chinese pharmacopoeia using a more complex traditional process (heated-reflux extraction process with methanol).

Doldolova et al. [65] studied the curcumin and antioxidant extraction capacity of five different DESs from turmeric (*Curcuma longa*) utilizing microwave-assisted extraction. It is important to note that polyphenols are not the only antioxidants present in turmeric, as several other non-phenolic compounds also exhibit antioxidant activity [95]. Herein, five DESs were prepared; fructose:ChCl:water (molar ratio of 2:5:5), sucrose:ChCl:water (1:4:4), fructose:lactic acid:water (1:5:5), sucrose:lactic acid:water (1:5:7), and lactic acid:ChCl:water (1:1:2), and referred as DES 1, 2, 3, 4, and 5. Different extraction parameters (e.g., extraction time, temperature, and solvent:solid ratio) were studied. The optimal extraction duration was 15–21 min at 64–71 °C, using 14–16 mL/0.2 g of dried sample. The differences in duration is due to different times being needed for different DES to penetrate and dissolve the curcumin in the turmeric. In this study, it is important to note that (1) despite DESs may degrade at high temperatures, there was no degradation observed, even at the harshest experiment conditions (75 °C for 30 min), and (2) higher solvent-solid ratios only produced a small increase in extraction efficiencies, even though it is known that higher ratios often enhance the mass transfer due to the higher concentration gradients. At the optimal conditions, all DESs except DES 1, presented a higher curcumin extraction yield and total antioxidants content than those obtained using 80% methanol:water solvent. DES 1, 2, 3, 4, 5, and 80% methanol:water curcumin yields were 13.95, 21.41, 24.81, 32.00, 23.06, and ca. 15 mg/g of dried sample, respectively. 

In a DES-based MAE recovery study, Pan et al. [66] obtained total phenolic and total flavonoid content from Lour (*Osmanthus fragrans*) flowers using a lactic acid:glucose (1:5) DES. An interesting aspect of this study is that optimal conditions for total phenolic and total flavonoid content extraction differ greatly. For example, the highest yield of flavonoids was obtained using a solid–liquid ratio of 30 mL g^−1^ under a microwave power of 500 W for 34.77 s. In contrast, the phenolic yield was only 80.7% at the same condition. The finding highlights that a specific process condition is needed to extract the desired compound effectively. Overall, the highest total phenolic and flavonoid yields were 192.20 ± 1.08 mg GAE g^−1^ and 560.13 ± 0.51 mg RE g^−1^, respectively. 

Use of DESs in biomass processing is a growing research field that aims to develop green and efficient methods for biomass valorization. Food industry waste represents a great opportunity for biomass valorization due to the large volumes of residues and by-products, i.e., ~28–35 Mt of orange peel waste (by-product) are produced worldwide, where only a small amount is reused as animal feed, fertilizer, biofuel, and bioactive compounds extraction and the majority is disposed of as land-filling or compost [96]. Concerning this, Panić et al. [67] proposed the use of DESs for valorization of orange peel waste by (*R,S*)-1-phenylethyl acetate hydrolyzation, as well as D-limonene, protein and polyphenol extraction. From preliminary experiments in an integrated bio-refinery protocol, the authors concluded that the ChCl:ethylene glycol (1:2) solvent with 50% water (w/w) was the most efficient out of seven prepared DESs. In the experiment, 10 mL of the selected DES, 0.015 g L^−1^ (*R,S*)-1-phenylethyl acetate, and 0.2 g mL^−1^ orange peel were mixed and placed in a shaker at 850 rpm for seven days. Later, using a glass column with a Sepabeads SP825L porous resin, the polyphenols were separated from the solvent. Using the best DES, a polyphenol yield of 45.7 mg g^−1^ was obtained with good limonene (0.5 mg g^−1^) and protein (7.7 mg g^−1^) extraction yields. The DES also showed high enantio-selectivity in the hydrolysis of (*R,S*)-1-phenylethyl acetate.

Additionally, aiming at bio-refinery processing in the food industry, López-Linares et al. [68] evaluated the phenolic extraction from brewer’s spent grain using a DES-based MAE. First, four DESs (ChCl:ethylene glycol, ChCl:lactic acid, ChCl:glycerol, and ChCl:1,2-propanediol with a 1:2 molar ratio and 25% water (*v/v*)) were screened in experiments with a maximum microwave power of 700 W, at 65 °C for 20 min. From the initial screening, the ChCl:glycerol solvent proved to be the most efficient, obtaining 2.3 mg GAE g^−1^, higher than the obtained value of 1.2 mg GAE g^−1^ using methanol:water (80:20 *v/v*) solvent. Additionally, the process extracted not only phenolic compounds but also lignin, with delignification ranging from 0.13 to 20.75%, adding to its advantages for bio-refinery use. Later, variations of temperature (50–100 °C), time (5–25 min), and water percentage in the ChCl:glycerol solvent (20–70%) were studied for process optimization. As expected, an increase in temperature raised the phenolic extraction yield due to the improved phenolic solubility and diffusivity in the solvent. However, low extraction yields were obtained at high temperatures using high-water percentage solvents, believed to be caused by a loss of eutectic properties in the solvent due to the rupture of hydrogen bonds. The highest yield obtained in this MAE process was with 37.46% (*v/v*) water in the ChCl:glycerol DES at 100 °C for 13.3 min, generating a total phenolic yield of 2.89 mg GAE g^−1^. This study confirms that a fast, greener and more efficient phenolic extraction method from brewer’s spent grain is possible using this DES. Furthermore, this process presents a delignification capacity and a low sugar concentration, displaying its potential in bio-refinery processing.

Lignin, an underutilized high-molecular mass phenolic biopolymer [97], is the most abundant aromatic polymer and the biggest source in the world [98,99]. Currently, extensive research is being done to replace fossil fuels with renewable energy sources, such as lignin [100,101]. Extraction of lignin from maritime pine (*Pinus pinaster*) wood using several pure and aqueous DES mixtures was reported by Fernandes et al. [69]. The experiments were performed at an extraction temperature of 150 °C for 2 h with a solid-solvent ratio of 1:10. It was observed that ChCl-based DESs presented a higher lignin extraction yield than that betaine or urea-based DESs. For binary DESs, lactic acid:ChCl with a 5:1 molar ratio was the most efficient, extracting a lignin yield of ca. 40% with a purity of 68.3%; nevertheless, the tartaric acid:ChCl (1:2) DES produced the best lignin purity of ca. 83%. It was observed that carboxylic acids containing alpha-hydroxy acids presented a higher affinity for lignin than linear carboxylic acids. The tertiary lactic acid:tartaric acid:ChCl (4:1:1) DES showed the best performance, surpassing all pure, binary and tertiary DESs, exhibiting a 92.7% and ca. 27% of lignin purity and extraction yield, respectively. Additionally, Fourier-transform infrared spectroscopy (FTIR) showed no significant differences in lignin structures regardless of the DES used. Furthermore, the extraction temperature and time were optimized using this particular solvent, showing the typical trend where the extraction yield increases with temperature. However, lignin purity decreased in extractions at over 150 °C (extractions carried out for 2 h). Concerning time, 1 h extraction time was sufficient for an adequate extraction at 175 °C, as there were no significant differences in extraction yield and lignin purity for 1, 2, and 4 h experiments. At optimal conditions with this DES, a 95 wt.% lignin recovery with an 89 wt.% purity was achieved. The DES was reused in 3 consecutive extractions, producing no significant differences in each extraction yield, thus proving its reusability.

Biomass pre-treatment is an essential step in bio-refineries, given the need to disrupt the lignocellulosic material before further processing [102]. In this aspect, DESs have risen as conventional solvent substitutes. Su et al. [70] studied a DES pre-treatment for poplar (*Populus*) sawdust where lignin removal was evaluated. ChCl:lactic acid DESs with different molar ratios (1:2, 1:4, 1:6, 1:8, and 1:10) were prepared, and the processes were carried out in a reaction vessel at either 110 or 130 °C, for 90 min with 10 g of poplar powder and 100 g of DES. The extracted lignin amount was significantly higher in the process at 130 °C, a phenomenon that was attributed to a viscosity and surface tension reduction in the solvent, which eases the solvent permeation into the poplar’s cell wall [103]. There was also a clear relationship between lignin removal and DES molar ratio, where varying the molar ratio from 1:2 to 1:10 increased lignin recoveries accordingly. The optimal extraction was observed at 130 °C with a 1:10 molar ratio DES, reaching a lignin removal up to 89.3%. This process provided selective lignin and xylan removal with high cellulose preservation, making it a very efficient alternative to the conventional solvent pre-treatment of lignocellulosic biomass. 

**Figure 4:**
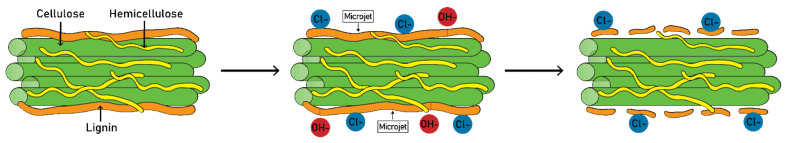
Process mechanism of ultrasound-assisted NaOH-aqueous DES pre-treatment. Adapted from Ong et al. [71].

Ong et al. [71] explored delignification of oil palm (*Elaeis guineensis)* frond using an ultrasound-assisted NaOH-aqueous DES pre-treatment. Currently, the pre-treatment processes represent ~20–48% of the total operational cost in bio-refineries, and this is mainly due to the higher fractionation energy required due to the recalcitrant nature of lignocellulosic biomass. UAE is recognized as a powerful technology for phytochemical extraction and easily applied on small and large scales [81]. A ChCl:urea (1:2 molar ratio) DES was mixed with 4-parts of water, obtaining a 1:4 water:DES aqueous mixture, followed by the addition of 0.1, 0.5, 1.5, 2.5, and 3.5% (*w/v*) NaOH to produce the final NaOH-aqueous DESs. Pure and aqueous DESs with NaOH were used as references. The optimum conditions; NaOH concentration of 2.5%, an ultrasound amplitude of 60%, and extraction duration of 30 min, produced a lignin removal of 47.00 ± 0.16%. The authors proposed a working mechanism where the ultrasound-produced microbubbles in the low viscosity NaOH-DES solvent forming micro-jet streams directed towards the biomass surfaces, disintegrating and concurrently removing the hydrophobic wax layer from the frond’s surfaces. The cavitation approach as a destructive force is already proven in several applications, confirming the above-mentioned proposed mechanism [104]. These micro-jets also improved the solvent penetration into the biomass, by inducing the frond fibers swelling and facilitating the solvent penetration. On the other hand, the high NaOH basicity increased the lignin solubility to achieve a high lignin removal. The process mechanism is depicted in Figure 4. 

Several other studies also showed the DES-based UAE extraction potential. Fu et al. [72] developed a DES-coupled pulse-UAE for anthocyanin recovery from blueberry (*Vaccinium* spp.) pomace. Anthocyanins, the biggest group of phenolic pigments with relevant antioxidant activity, are compounds of interest in the food industry due to their biological activities and natural colorants [105]. Total anthocyanins content in blueberry pomace was determined by Giusti and Wrolstad [106] and expressed in mg of Cyanidin-3-*O*-glucoside equivalent (C3GE) per gram of sample. First, seven ChCl-based DESs with different carboxylic acids and polyalcohols as the HBDs. Given the high pure DES viscosities, 10, 30, and 50% DES-water mixtures were produced. The selection for the highest extraction efficiency was conducted using the conventional heat-assisted extractions, and the total extracted anthocyanin contents was compared with the obtained values using water and ethanol. The 30% DES-water mixtures obtained the highest yields. As discussed earlier, water addition into DESs reduces the solvent viscosity, easing the mass transfer of the targeted compound from the source to the solvent; however, when excessive water is added, the DES structure is dismantled causing a reduction in the extraction yield [3,43,89]. It was noted that DESs with carboxylic acids as HBD showed higher extraction efficiencies than those with polyalcohols. This is ascribed to the higher polarity and lower pH value of the carboxylic acid-based DESs, as anthocyanins are highly polar compounds found frequently in their flavylium cation form and stable at pH values below 2 [107]. It was also observed that higher density and lower viscosity in the DES corresponded to a higher extraction of total anthocyanin content. The highest extraction yield was obtained with the ChCl:oxalyc acid DES (1:1 molar ratio) with 30% water, outperforming extractions using ethanol and water. Using the best DES, four different UAE parameters were then assessed, including ultrasonic time, ultrasonic power, temperature, and solvent to solid ratio. The optimum process conditions were: an ultrasonic time of 3.2 min, with 325 W power at 76 °C, and a solvent to solid ratio of 60 mL g^−1^, acquiring a total anthocyanin yield of 24.27 ± 0.05 mg C3GE g^−1^.

Chen et al. [73] documented phenolic extraction from lily (*Lilium lancifolium* Thunb.) through DES-based UAE. In this study, four DESs were synthesized using ChCl as the HBA and four different HBDs: ethylene glycol, lactic acid, glycerol, and formic acid, each at a molar ratio of 1:2. The authors utilized 1 g of *Lilium lancifolium* in each extraction, obtaining the ChCl:ethylene glycol DES as the most efficient. Additionally, the effects of water content in the solvent, solid-solvent ratio, temperature, and extraction time were also studied. It was observed that water addition from 10 to 30% increased the extraction rate, with 20% water being the best, whereas 40 to 50% water addition was detrimental to the extraction. The optimum conditions were: 50 °C, a 40 min extraction with a solid-solvent ratio of 1:25. At these optimal conditions, yields of 0.31 ± 0.06, 0.29 ± 0.03, and 3.04 ± 0.38 mg g^−1^ for regaloside B, regaloside C, and regaloside E were achieved, respectively. Compared to the conventional hot water extraction method, higher yields and shorter extraction times were obtained with DES.

Mansinhos et al. [74] carried out phenolic compounds separation from *Lavandula pedunculata* subsp. *lusitanica* with ten different DESs using UAE. *Lavandula pedunculata* is known to have medicinal properties, credited partly to its phenolic content [108]. Mansinhos et al. used four HBAs (glycerol, glucose, ChCl, and proline) with different HBDs (citric acid, urea, lactic acid, xylitol, in their work and malic acid). After a preliminary evaluation, proline:lactic acid (1:1) DES presented the highest total phenolic amount (56.00 ± 0.77 mg GAE g^−1^ DW), in which the most abundant extracted compounds were rosmarinic acid, ferulic acid, and salvianolic acid. The results were in agreement with the previously reported phenolic compositions in *Lavandula* species [109]. It is important to mention that the individual phenolic yield was different for each DES, indicating an opportunity for selective phenolic extraction.

In a study by Jakovljević et al. [75], carnosic acid and carnosol extraction from sage (*Salvia officinalis*) was conducted through three extraction methods, i.e., conventional stirring and heating, UAE, and mechanochemical extraction (MCE), using DESs. Firstly, seventeen DESs with ChCl as the HBA were prepared with their respective 10, 30 and 50% aqueous mixtures and studied through stirring and heating extraction to obtain the best performing solvent. For carnosol extraction, the ChCl:1,4-butanediol (1:2) DES was the most effective, whereas the acidic ChCl:lactic acid (1:2) DES was the most efficient for carnosic acid extraction. This is attributed to the higher acidic DES polarity compared with the DESs with sugar HBDs. Further experiments were carried out with the ChCl:lactic acid (1:2) DES. It was found that the stirring and heating and UAE methods were the most efficient, believed to be due to the continuous stirring in the earlier method and the faster compound diffusion and increased mass transfer due to the cavitation effect in the latter. Nonetheless, MCE reduces the extraction time and solvent amount. Similar amounts of carnosol and carnosic acid were obtained in 90 min stirring and heating and UAE extraction processes, and only 3 min of the MCE process, with a minimal DES amount. This is caused by the glass beads that enhance the plant-DES mixing. The highest carnosic acid extraction yields were 14.43 ± 0.28 μg mg^−1^ for stirring and heating (10% of H_2_O, 90 min, 50 °C); 14.00 ± 0.02 μg mg^−1^ for UAE (10% of H_2_O, 60 min, 70 °C); and 8.26 ± 0.45 μg mg^−1^ for MCE (10% of H_2_O, 2 min, 5 m s^−1^ vibration speed). Moreover, carnosol and carnosic acid extraction with the ChCl:lactic acid (1:2) DES proved to be more efficient than conventional solvents such as water, ethanol, and 30–70% (*v/v*) water-ethanol aqueous mixtures.

Patil et al. [76] described a DES-based UAE of curcuminoids from turmeric. Herein, nine ChCl-based DESs were prepared and screened for curcuminoids extraction using glycerol (1:2), ethylene glycol (1:1), 1,4-butanediol (1:3), lactic acid (1:1), malic acid (1:1), citric acid (2:1), xylose (1:1), glucose (2:1), and fructose (2:1) as the HBDs. The UAE was carried out with sonication at 22 kHz frequency and 200 W of power. The highest curcuminoids extraction yield (ca. 58.87 mg g^−1^) was obtained with the ChCl:Lactic acid DES, which showed the highest curcuminoid solubility (13.7 mg mL^−1^) during a solubility test. Hence, process optimization was performed with this DES. The highest extraction yields were obtained using the acid-based DESs, followed by sugar-based DESs, while the alcohol-based DESs presented the lowest yields. In the optimization, increasing the DES molar ratio from 1:1 to 1:4 provided a higher curcuminoid yield; however, there were no significant differences. Thus, the 1:1 ratio was maintained as a larger HBD amount is not cost-effective. Water addition to the DESs was also studied, and 20% water content presented the highest yield, attributed to a better dissipation of ultrasonic energy and cavitation enhancement. The authors also examined the turmeric loading and particle size, where 5% turmeric loading presented the highest yield, at higher loadings a constraint in mass transfer within the cluster of particles was observed [110]. The particle size of 0.355 mm was chosen due to higher aspect-ratio in smaller particles (higher particle cells in contact with solvent), but a further size reduction promoted particle agglomeration and sedimentation. Using the optimal conditions (intensity of 70.8 W cm^−2^, pulse mode of 60% duty cycle at 30 °C for 20 min), a 77.13 mg g^−1^ curcuminoid yield was obtained. In a comparative study between ethanol-based Soxhlet extraction and DES-based batch extraction, UAE presented a much lower energy requirement and a yield higher than the batch extraction. Interestingly, the yield is similar to the Soxhlet extraction but only consumed 0.67% of the energy required in the Soxhlet process. 

Very recently, Ünlü [77] developed a phenolic extraction from the olive leaf through UAE with DESs, expressed as total polyphenol and flavonoid yields. Like many other natural sources presented in this review, olive leaf has been utilized for medicinal purposes since ancient times due to its beneficial antioxidant, antibacterial, and antifungal properties [111]. Eight DESs were prepared and evaluated for total polyphenol and flavonoid yields. Before optimization, the glucose:sucrose:water (1:1:11) solvent was screened to be the most efficient for total polyphenol extraction, yielding around 20.49 ± 0.50 mg GAE g^−1^, ascribed to the low pH compared to the other sugar-based DESs prepared. For the total flavonoid content, the highest yield was obtained with the ChCl:lactic acid (1:2) DES, achieving 8.44 ± 0.30 mg ApE g^−1^, thanks to its low viscosity. Afterwards, the experimental conditions were optimized, and both polyphenol and flavonoid content were extracted using a ChCl:fructose:water (5:2:5) DES (42.69% water content, liquid-to-solid ratio of 40.66 mL g^−1^, 75 °C, 1 h UAE at 140 W and 37 kHz) obtaining 187.31 ± 10.3 mg GAE g^−1^ and 12.75 ± 0.6 mg ApE g^−1^ for polyphenols and flavonoids, respectively.

Using eleven different DESs in a UAE study, Ivanović et al. [78] extracted phenolics from *Helichrysum arenarium* L. inflorescences. The maximum phenolic contents (determined by spectrophotometry) of 30.15 ± 0.42, and 29.75 ± 0.49 mg g^−1^ were achieved using a ChCl:1,2-propanediol (1:2) and a ChCl:1,4-butanediol (1:6) 25% water DESs, respectively. These yields were higher than those obtained with processes carried out with 80% methanol and water as solvents under the same conditions. Furthermore, since the phenolic compounds were quantified individually, the authors determined that the high yields were attributed to the less polar phenolic compounds, such as secoiridoids and flavonoids. Similar findings were also previously reported by Garcia et al. [87].

Olive oil presents an important amount of phenolic compounds. It contains at least 36 structurally different phenolic compounds, and studies have proven that olive oil’s phenolic compounds display positive effects on human health [112]. Rodríguez-Juan et al. [79] extracted phenolic compounds from virgin olive oil with a ChCl:xylitol aqueous DES through agitation in a water bath, followed by solvent removal using a XAD-16 resin column. The DES was washed with water in the column, and the phenolics were eluted with ethanol, thus recycling the DES. The reported phenolic content was the sum of specific compounds quantified by HPLC (e.g., hydroxytyrosol, tyrosol, 1-acetoxypinoresinol, 3,4-DHPEA-EDA, etc.). Different test conditions were explored; a temperature of 30–90 °C, 0.5–6 h of extraction time, and different virgin olive oil:DES ratios (1:1, 1:3, 1:4, 1:7). It was observed that the most efficient phenolic extraction conditions were at 40 °C for 1 h using an oil:DES ratio of 1:1, obtaining a yield of 555.36 ± 21.93 mg of phenolic per kg of oil.

**Figure 5:**
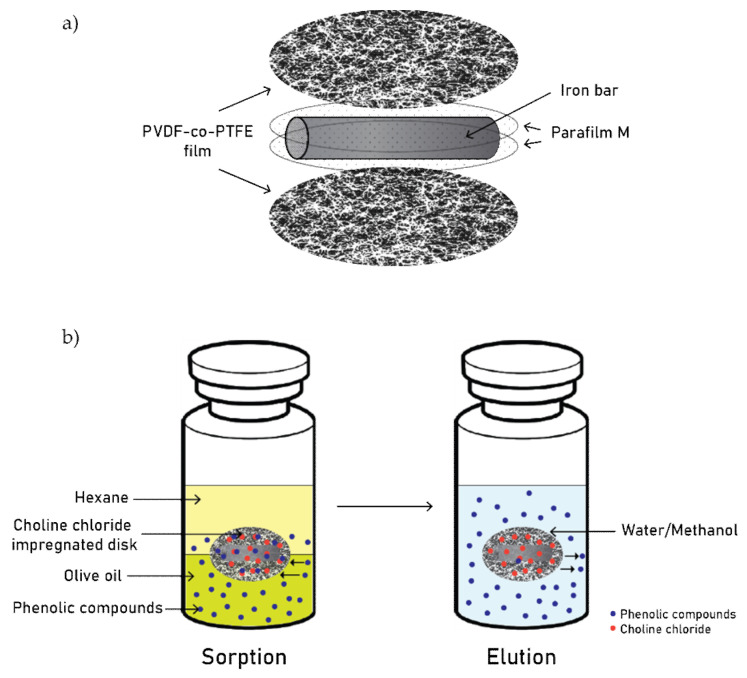
Rotating disk sorptive extraction: disk composition (**a**), sorptive extraction and elution process (**b**). Adapted from Shishov et al. [80].

Shishov et al. [80] investigated phenolic extraction from olive oil using a rotating disk sorptive extraction method, where the extraction mechanism relies on in situ DES formation between a ChCl-coated rotating disk and the phenolic compounds in the oil sample. The rotating disk was made of two polymer films, sandwiching a Parafilm M-wrapped iron wire for magnetic stirring (see the configuration in Figure 5a). After the assembly, the disk was clamped with glass and heated at 60 °C for 15 min, followed by a coating with 10 μL of ChCl solution. The rotation disk was placed inside a vial consisting of 1 mL of olive oil and 1 mL of n-hexane (used to reduce the oil’s viscosity). After a period of stirring, the disk was removed, and the n-hexane was evaporated before placing the disk into a vial with 200 μL of a water-ethanol mixture (2:1 *v/v*). In this stage, the phenolic compounds were separated from the DES due to DES decomposition (ChCl dissolves in water and disintegrates from the DES formation, and methanol enhances phenolic solubility). The extraction process is illustrated in Figure 5b. The optimal extraction and elution conditions were studied, and the authors found that poly(vinylidene fluoride-co-tetrafluoroethylene) (PVDF-co-PTFE) films presented a higher absolute extraction recovery than cellulose acetate, nylon and polyethersulfone films. This is due to its high hydrophobicity, a crucial characteristic that allows PVDF-co-PTFE to be more oil-selective. As for the oil dilution, no significant differences were found between n-hexane, n-heptane, and isooctane; however, n-hexane was chosen for its high volatility, thus reducing the evaporation time. A concentration of 300 g L^−1^ ChCl was chosen as it provided the highest absolute extraction recovery. As for the extraction conditions, chemical equilibrium was reached at 15 min at 150 rpm rotation. The optimal elution volume and time were 0.2 mL and 2 min, respectively. Absolute extraction recoveries of gallic acid, protocatechuic acid, tyrosol, vanillic acid, *p*-coumaric acid, syringaldehyde and thymol were 87, 79, 76, 75, 82, 81, and 66%, respectively. The extraction performances were comparable to the obtained values in a methanol-water (60:40 *v/v*) extraction method. Using specific techniques, such as FTIR and scanning electron microscope (SEM), the authors confirmed ChCl disk-impregnation, as well as DES formation and retention. The FTIR analysis confirmed that the O-H absorption peak shifted after ChCl coating and after ChCl-phenolic compound interaction, a change believed to be due to hydrogen bond formation. The spectra also indicated several peaks associated with the phenolic compounds’ functional groups, confirming its extraction into the rotating disk. The SEM images showed a fibrous pristine disk structure, which smoothed out after ChCl impregnation and later presented a different structure after phenolic uptake. This study offers a novel, rapid extraction and elution method, utilizing an in situ synthesized DES with the phenolic compounds from the sample source. 

## 4. Concluding Remarks

The DES-based phenolic extraction research has increased exponentially due to its apparent advantages over conventional solvents. As presented, DESs are catalogued as green alternatives, given their biodegradability and reusability; moreover, numerous DES components are non-toxic, and some are even safe for human consumption, further broadening their industrial applicability. DESs have shown the ability to preserve phenolic compounds in solutions. These properties make DESs excellent candidates in the industry not only for extractive processes but also as their final carrier eliminating the need for further processing, as suggested by Skarpalezos and Detsi [113]. In addition to the intrinsic characteristics such as low volatility and melting point, good thermal and chemical stability, and low cost, their advantages are very attractive not only in biomolecule extraction or separation but also in biomass waste revalorization through DES-based bio-refinery processes.

DES selection and optimum process conditions are vital for an adequate extraction. It is important to note that there are limitless DES components, leading to a whole new variety of DESs (with different physicochemical properties) that may present enormous opportunities for more selective extractions. Likewise, extraction method selection will vary depending on the compound of interest and its source. More importantly, DESs have demonstrated their ability to assist the emerging techniques and protocols in the phenolic compound separation. Many studies have shown the great DES performances in biomolecule extractions, displaying comparable or higher yields than conventional solvents. More industrial applicability research is encouraged and required, given its potential to reduce operation costs and decrease operational risks for workers, consumers, and the environment. However, as it can be observed in this review, the screening for an adequate DES is a time-consuming process, hence, several authors encourage the use of computational tools for DES extraction efficiency modeling [114], which is increasingly applied by researchers along with the extraction experiments.

DES components are often non-volatile. Thus, phenolic separation from the extract can be more challenging than the classic organic solvents (simple solvent evaporation will not be effective in DESs). In the reviewed studies, these aspects are often not being addressed accordingly. In our opinion, (1) new DES application study (for precious compound extraction) should include the extracted component separation, and (2) the DES advantages compared to the conventional solvents should be proved appropriately and may include a brief economic assessment. The whole process cost can be strongly affected by the DES components and the final phenolic separation from DES. Finally, the DES recycling possibility should also be addressed.

## Figures and Tables

**Table 1:** Classification of DES. Adapted from [48].

Type	Formula	Terms
Type I	Cat^+^X^−^zMCl_x_	M = Zn, Sn, Fe, Al, Ga, In
Type II	Cat^+^X^−^zMCl_x_∙yH_2_O	M = Cr, Co, Cu, Ni, Fe
Type III	Cat^+^X^−^zRZ	Z = CONH_2_, COOH, OH
Type IV	MCl_x_ + RZ = MCl_x_−_1_^+^∙RZ + MCl_x+1_^−^	M = Al, Zn Z = CONH_2_, OH

**Table 2:** Phenolic compound extraction processes presented in this review.

Source	DES Used (Molar Ratio)	Co-Solvent	Extraction Method and Conditions	Yield	Reference
Manuka leaves (*Leptospermum scoparium*)	ChCl:Lactic acid (1:2)	-	Heating and stirring Temperature: 50 °C Extraction time: 67 min Liquid-solid ratio: 100:5.07	TPC: 59.82 mg GAE g^−1^	[57]
*Polygonum cuspidatum* root	TBAC:Ethylene glycol (1:3)	40% water 4.5% HCl	Heating and stirring Temperature: 80 °C Extraction time: 80 min Rotational speed: 500 rpm Liquid–solid ratio: 40:1	Resveratrol: 12.26 ± 0.14 mg g^−1^	[58]
*Buddleja globosa* Hope leaves	ChCl:Lactic acid (1:1)	20% water	Heating and stirring Temperature: 60 °C Extraction time: 50 min Rotational speed: 340 rpm Liquid–solid ratio: 100:1	TPC: ca. 175 mg GAE g^−1^ Luteolin 7-O-glucoside: ca 4.1 mg g^−1^ Verbascoside: ca. 39 mg g^−1^	[59]
	L-proline:Citric acid (1:1)	20% water		TPC: ca. 175 mg GAE g^−1^ Luteolin 7-O-glucoside: ca. 4 mg g^−1^ Verbascoside: 51.045 mg g^−1^	
Rosemary leaves (*Rosmarinus officinalis* L.)	ChCl:1,2-propanediol (1:2)	50% water	Heating and stirring Temperature: 65 °C Extraction time: 15 min Rotational speed: 600 rpm Liquid–solid ratio: 40:1	TPC: 78 mg GAE g^−1^	[60]
Black mustard (*Brassica nigra* L.) seed	TEOA:Glycerol (1:2)	25% ethanol	Heating and stirring Temperature: 65 °C Extraction time: 180 min Rotational speed:1000 rpm Liquid–solid ratio: 10:1	TPC: 32.2 ± 0.2 mg GAE g^−1^ TFC: 7.2 ± 0.2 mg QE g^−1^	[61]
	TEOA:Propylene glycol (1:2)	25% ethanol		TPC: 29.3 ± 0.3 mg GAE g^−1^ TFC: 7.4 ± 0.3 mg QE g^−1^	
Coffee	L-serine:Lactic acid (1:4)	30% methanol	Two-phase hollow fiber-liquid microextraction (HF-LPME) Fiber material: polypropylene HF DES immersion time: 30 s Rotational speed: 840 rpm	Caffeic acid: 0.032 µg g^−1^	[62]
Green Tea				Caffeic acid: 0.022 µg g^−1^	
Tomato				Caffeic acid: 0.012 µg g^−1^	
Green tea	ChCl:ethylene glycol (1:2)	29% water	Heating and stirring Temperature: 84 °C Extraction time: 39 min Liquid–solid ratio: 44:1	Phenolic extraction yield: 20.12%	[63]
Radix et Rhixoma Rhei	1- tetradecanol:10-undecenoic acid (1:4)	10% HCl	Heating and stirring Temperature: 67 °C Extraction time: 20 min Rotational speed: 500 rpm Liquid–solid ratio: 12:1	Anthraquinones: 21.52 mg g^−1^	[64]
Turmeric (*Curcuma longa)*	Sucrose:Lactic acid:Water (1:5:7)	-	Microwave-assisted extraction Temperature: 68.2 °C Extraction time: 15.4 min Maximum power: 1500 W Liquid–solid ratio: 14.5:2	Curcumin: 32.00 mg g^−1^	[65]
Lour (*Osmanthus fragrans*) flower	Lactic acid:glucose (1:5)	85% water	Microwave-assisted extraction Extraction time: 0.5 s Power: 500 W Liquid–solid ratio: 30:1	TPC: 192.20 ± 1.08 mg GAE g^−1^ TFC: 560.13 ± 0.51 mg RE g^−1^	[66]
			Microwave-assisted extraction Extraction time: 60.00 s Power: 500 W Liquid–solid ratio: 38.41:1	TPC: 238.09 ± 0.81 mg GAE g^−1^ TFC: 321.07 ± 1.65 mg RE g^−1^	
Orange peel waste	ChCl:Ethylene glycol (1:2)	50% water	Stirring Extraction time: 7 days Rotational speed: 850 rpm Liquid–solid ratio: 5:1	Polyphenols: 45.7 mg g^−1^	[67]
Brewer’s spent grain	ChCl:Glycerol (1:2)	37.46% water	Microwave-assisted extraction Temperature: 100 °C Extraction time: 13.3 min Maximum power: 700 W	TPC: 2.89 mg GAE g^−1^	[68]
Maritime Pine (*Pinus pinaster*) wood	Lactic acid:Tartaric acid:ChCl (4:1:1)	-	Heating and stirring Temperature: 175 °C Extraction time: 90 min Liquid–solid ratio: 10:1	Lignin removal: 94.9 ± 3.3%	[69]
Poplar (*Populus*) sawdust	ChCl:Lactic acid (1:10)	-	Heating and stirring Temperature: 130 °C Extraction time: 90 min Liquid–solid ratio: 10:1	Lignin removal: 89.3%	[70]
Oil palm (Elaeis guineensis) frond	ChCl:Urea (1:2)	20% water 2.5% NaOH	Ultrasound-assisted extraction Extraction time: 30 min Ultrasound amplitude: 60%	Lignin removal: 47.00 ± 0.16%	[71]
Blueberry (*Vaccinium* spp.) pomace	ChCl:Oxalic acid (1:1)	30% water	Pulse- Ultrasound-assisted extraction Temperature: 76 °C Extraction time: 3.2 min Power: 325 W Liquid–solid ratio: 60:1	Total anthocyanin yield: 24.27 ± 0.05 mg C3GE g^−1^	[72]
Lily (*Lilium lancifolium* Thunb.)	ChCl:Ethylene glycol (1:2)	20% water	Ultrasound-assisted extraction Temperature: 50 °C Extraction time: 40 min Liquid–solid ratio: 25:1	Regaloside B: 0.31 ± 0.06 mg g^−1^ Regaloside C: 0.29 ± 0.03 mg g^−1^ Regaloside E: 3.04 ± 0.38 mg g^−1^	[73]
*Lavandula pedunculata* subsp. *lusitanica*	Proline:Lactic acid (1:1)	30% water	Ultrasound-assisted extraction Extraction time: 60 min Frequency: 37 kHz	TPC: 56.00 ± 0.77 mg GAE g^−1^	[74]
Sage (*Salvia officinalis*)	ChCl:Lactic acid (1:2)	10% water	Stirring and heating Temperature: 50 °C Extraction time: 90 min	Carnosic acid: 14.43 ± 0.28 μg mg^−1^ Carnosol: 4.83 ± 0.09 μg mg^−1^	[75]
	ChCl:Lactic acid (1:2)	10% water	Ultrasound-assisted extraction Temperature: 70 °C Extraction time: 60 min	Carnosic acid: 14.00 ± 0.02 μg mg^−1^ Carnosol: 4.18 ± 0.05 μg mg^−1^	
	ChCl:Lactic acid (1:2)	10% water	MCE Extraction time: 2 min Vibration speed: 5 m s^−1^	Carnosic acid: 8.26 ± 0.45 μg mg^−1^ Carnosol: 1.87 ± 0.33 μg mg^−1^	
Turmeric (*Curcuma longa*)	ChCl:Lactic acid (1:1)	20% water	Ultrasound-assisted extraction—direct sonication Temperature: 30 °C Extraction time: 20 min Frequency: 22 kHz Intensity: 70.8 W cm^−2^ Pulse mode: 60% Particle size: 0.355 mm Liquid–solid ratio: 20:1	Curcuminoids: 77.13 mg g^−1^	[76]
Olive leaf	ChCl:Fructose:Water (5:2:5)	42.69% water	Ultrasound-assisted extraction Temperature: 75 °C Extraction time: 60 min Power: 140 W Frequency: 37 kHz Liquid–solid ratio: 40.66:1	TPC: 187.31 ± 10.3 mg GAE g^−1^ TFC: 12.75 ± 0.6 mg ApE g^−1^	[77]
*Helichrysum arenarium* L. inflorescences	ChCl:1,2-propanediol (1:2)	25% water	Ultrasound-assisted extraction Temperature: 50 °C Extraction time: 60 min	Phenolics: 30.15 ± 0.42 mg g^−1^	[78]
	ChCl:1,4-butanediol (1:6)			Phenolics: 29.75 ± 0.49 mg g^−1^	
Olive oil	ChCl:Xylitol:Water (2:1:3)	-	Heating and stirring Temperature: 40 °C Extraction time: 60 min Liquid:oil ratio: 1:1	Phenolics: 555.36 ± 21.93 mg kg^−1^	[79]
Olive oil	ChCl:Extracted phenolics	-	Rotating disk sorptive extraction Extraction time: 15 min Disk material: poly(vinylidene fluoride-co-tetrafluoroethylene) Rotational speed: 150 rpm ChCl concentration: 300 g L^−1^	Absolute extraction recovery Gallic acid: 87% Protocatechuic acid: 79% Tyrosol: 76% Vanillic acid: 75% *p*-coumarinic acid: 82% Syringaldehyde: 81% Thymol: 66%	[80]

## Data Availability

Data are contained within the article.

## Sample Availability

Samples of the compounds are not available from the authors.
